# Prognostic and therapeutic value of disruptor of telomeric silencing-1-like (DOT1L) expression in patients with ovarian cancer

**DOI:** 10.1186/s13045-017-0400-8

**Published:** 2017-01-23

**Authors:** Xiaoxue Zhang, Dan Liu, Mengchen Li, Canhui Cao, Dongyi Wan, Bixin Xi, Wenqian Li, Jiahong Tan, Ji Wang, Zhongcai Wu, Ding Ma, Qinglei Gao

**Affiliations:** Cancer Biology Research Center (Key Laboratory of the Ministry of Education), Tongji Hospital, Tongji Medical College, Huazhong University of Science and Technology, Wuhan, People’s Republic of China

**Keywords:** DOT1L, Ovarian cancer, Prognosis, ChIP-seq, G1 arrest

## Abstract

**Background:**

Epigenetics has been known to play a critical role in regulating the malignant phenotype. This study was designed to examine the expression of DOT1L (histone 3 lysine 79 methyltransferase) and H3K79 methylation in normal ovarian tissues and ovarian tumors and to explore the function of DOT1L and its underline mechanisms in ovarian cancer.

**Methods:**

The expression of DOT1L and H3K79 methylation in 250 ovarian tumor samples and 24 normal ovarian samples was assessed by immunohistochemistry. The effects of DOT1L on cell proliferation in vitro were evaluated using CCK8, colony formation and flow cytometry. The DOT1L-targeted genes were determined using chromatin immune-precipitation coupled with high-throughput sequencing (ChIP-seq) and ChIP-PCR. Gene expression levels were measured by real-time PCR and immunoblotting. The effects of DOT1L on tumor growth in vivo were evaluated using an orthotopic ovarian tumor model.

**Results:**

DOT1L expression and H3K79 methylation was significantly increased in malignant ovarian tumors. High DOT1L expression was associated with International Federation of Gynecology and Obstetrics (FIGO) stage, histologic grade, and lymphatic metastasis. DOT1L was an independent prognostic factor for the overall survival (OS) and progression-free survival (PFS) of ovarian cancer, and higher DOT1L expression was associated with poorer OS and PFS. Furthermore, DOT1L regulates the transcription of G1 phase genes CDK6 and CCND3 through H3K79 dimethylation; therefore, blocking DOT1L could result in G1 arrest and thereby impede the cell proliferation in vitro and tumor growth in vivo.

**Conclusions:**

Our findings first demonstrate that DOT1L over-expression has important clinical significance in ovarian cancer and also clarify that it drives cell cycle progression through transcriptional regulation of CDK6 and CCND3 through H3K79 methylation, suggesting that DOT1L might be potential target for prognostic assessment and therapeutic intervention in ovarian cancer.

**Electronic supplementary material:**

The online version of this article (doi:10.1186/s13045-017-0400-8) contains supplementary material, which is available to authorized users.

## Background

Ovarian cancer (OC) is the fourth most common cause of cancer-related deaths in women, with an estimated 200,000 cases and 125,000 deaths occurring annually worldwide [1, 2]. Despite current advances in surgery and adjuvant chemotherapy, the clinical outcome of patients with OC remains uncertain, with the 5-year survival rate being ~30% for advanced-stage disease. The poor survival rate is largely attributed to the poor understanding of the OC initiation and progression [3]. The discovery of novel biomarkers with high sensitivity and specificity for the early diagnosis and target therapies of OC will help to improve the prognosis of this disease.

Epigenetic changes, such as DNA methylation and histone post-translational modifications, are major contributing factors for carcinogenesis [4]. Given that epigenetic changes are reversible, there is a hope that reversing epigenetic malfunction in cancer cells could render them normal, launching a new and rapidly expanding field of cancer epigenetic therapeutics [5]. Disruptor of telomeric silencing-1-like (DOT1L), a human homologue of DOT1 in yeast, is the only histone methyltransferase that produces methylated H3K79 (H3K79me_*x*_, *x* = 1, 2, or 3). Globally, H3K79 methylation is enriched in actively transcribing genes [6]. In addition to transcription, Dot1 plays an important role in cell cycle regulation and the DNA damage response [7]. Recent studies have revealed that DOT1L is involved in the initiation and maintenance of mixed lineage leukemia (MLL)-rearranged leukemia, by associating with MLL fusion partner proteins, e.g., AF4, AF9, AF10, or ENL, resulting in abnormal H3K79 methylation and overexpression of a number of MLL target genes (e.g., HoxA9 and Meis1) [8–10]. Consequently, DOT1L is a drug target for MLL-rearranged leukemia [11, 12]. Furthermore, a few recent publications have reported that DOT1L plays crucial roles not only in leukemia, but also in solid tumors, such as breast cancer, esophageal squamous cell carcinoma, colorectal cancer, prostate cancer, and gastric cancer [13–19]. Moreover, DOT1L inhibitors have shown effective suppression of proliferation, migration, and invasion of breast cancer [20].

To date, the potential role of DOT1L and its malignancies in OC have not been assessed. In this study, the prognostic significance of DOT1L expression in OC for clinicopathological parameters and patient survival was evaluated. Moreover, it is very significant that we identified DOT1L as a promising therapeutic target for OC both in vitro and in vivo experiments.

## Methods

### Patients and samples

A total of 274 formalin-fixed paraffin-embedded tissue samples were included in this study, comprising 24 normal ovarian tissues, 15 benign ovarian tumor, 7 borderline ovarian tumor, and 228 malignant ovarian tumor samples. These samples were obtained from patients diagnosed with ovarian carcinoma who underwent intitial surgery at the Gynecology Department of Huazhong University of Science and Technology Affiliated Tongji Hospital from 2003 to 2013. The tissue samples were obtained with informed consent from patients. None of the patients had any prior history of chemoradiation, radiation, or hormonal therapy before the surgery. Clinical information of the patients’ was recorder in detail, and the diagnoses were confirmed by at least two pathologists. The histopathologic subtypes of the 7 borderline ovarian tumor and 228 malignant ovarian tumor samples included 211 serous cystadenocarcinomas, 5 mucinous cystadenocarcinomas, 12 endometrioid adenocarcinomas, 4 ovarian germ-cell tumors, 2 ovarian clear-cell carcinomas, and 1 ovarian transitional cell carcinoma. There were 20 cases in stage I, 17 cases in stage II, 159 cases in stage III, 29 cases in stage IV, and 10 cases remained unknown, according to the International Federation of Gynecology and Obstetrics (FIGO) staging system. With regard to histological grading, 40 cases had grade 1, 58 cases had grade 2, 120 cases had grade 3, and 17 cases remained unknown. The median age of patients (16–80 years old) was 54 years old. Among the 235 OC tissues, the total of 206 cases has been followed up. The present study was with the approval and support of the Ethics or Institutional Review Board of Tongji Hospital.

### Immunohistochemistry

Formalin-fixed, paraffin-embedded tissue blocks were retrieved from the archive and were analyzed by IHC described previously [21]. In short, The Avidin-Biotin Complex (ABC) Vectastain Kit (Zsgb-Bio, Beijing, China) was used and the DOT1L polyclonal antibody (1:250; ab64077; Abcam), H3K79me2 polyclonal antibody (1:200; ab3594; Abcam), cyclin D3 antibody (1:100; ab63535; Abcam), and the CDK6 polyclonal antibody (1:500; GTX103992; GeneTex) were used as primary antibodies incubated with tissue sections (2 mm) after heat-induced epitope retrieval (10 mM sodium citrate buffer of pH 6.0), followed by incubation with a secondary antibody conjugated to peroxidase (1:100; Dako). Detection was performed using diaminobenzidine for 8 min, and the slides were counterstained with hematoxylin.

The scoring system that was used incorporated both the intensity of the scoring (0 = absent, 1 = weak, 2 = moderate, 3 = strong) and the percentage of positive tumor cells (0 = 0%, 1 = 1–25%, 2 = 26–50%, 3 = 51–75%, 4 = 76–100%). Points for the intensity and the percentage of staining were added and assigned as overall score according to Kamat and M. Caroline [22, 23]. Two investigators scored all slides for DOT1L and H3K79 methylation staining and followed the criterions of double-blind trials. Based on the overall score, the DOT1L and H3K79 methylation staining results were classified into low expression (≤4) and high expression (>4).

### Cells and transfection

Human epithelial ovarian cell lines SK-OV-3, TOV21G, OV90, and CaOV3 were purchased from the American Type Culture Collection (Manassas, VA, USA). SK-OV-3 and CaOV3 were cultured in McCoy’s 5A Medium (Boster, Wuhan, China), while TOV21G and OV90 in mixed MCDB105 Medium (Sigma-Aldrich Co., St Louis, MO, USA) and M199 Medium (Gibco, Invitrogen, Carlsbad, CA, USA) (1:1) supplemented with 10% fetal bovine serum (Gibco, Invitrogen, Carlsbad, CA, USA) and 1% penicillin/streptomycin at 37 °C in a humidified atmosphere of 5% CO_2_ and 95% air. The cells were transduced with CMV-Fluc-IRES-RFP lentiviral particles (GeneChem, Shanghai, China). RFP/luciferase-expressing cells were isolated by FACS and used in living imaging. ShDOT1L-1 and shDOT1L-2 shRNA lentiviral particles (GeneChem) were used to knock-down DOT1L expression, targeting 5′-CGGATCTCAAGCTCGCTAT-3′ and 5′-AGTGCTCGAATTGAGAGAA-3′, respectively. ShCON, the short hairpin RNA (shRNA) not targeting any known gene, was used as control. SK-OV-3 and TOV21G cells were transduced with DOT1L shRNA lentiviral particles, or control shRNA lentiviral particles. The cells with stable transfection were selected with puromycin.

### Assay of gene expression by real-time RT-PCR

Total RNA was isolated using Trizol (Invitrogen, Carlsbad, CA). RT-PCR analysis was carried out in a CFX96 Touch™ Real-Time PCR Detection system (Bio-Rad Laboratories, Inc., Hercules, CA, USA) and iQ™ SYBR®Green Supermix (Bio‑Rad Laboratories, Inc., Hercules, CA, USA), which contained 5 ng complementary DNA (cDNA) and 10 pM of each primer. The primer sequences used for PCR are shown in Additional file 1: Table S1. The relative messenger RNA (mRNA expression) levels were normalized to that of glyceraldehyde 3-phosphate dehydrogenase (GAPDH). The PCR protocol is as follows: denaturation at 95 °C for 10 min; 40 cycles of denaturation at 95 °C for 10 sec; annealing at 60 °C for 30 sec; and extension at 72 °C for 30 sec. Each sample was assayed in triplicate, and relative mRNA expression levels were calculated using the △△Ct method.

### Western blot analysis

Western blot assay was performed following the protocol as previously described [24, 25]. Briefly, the cells were harvested and lysed using ice-cold RIPA lysis buffer (50 mM Tris-HCl (pH 7.4), 150 mM sodium chloride, 1% Nonidet P-40, and 0.5% sodium deoxycholate). Following centrifugation at 10,000×*g* for 15 min at 4 °C, the proteins in the supernatants were quantified by Bradford method and separated using 10% SDS-PAGE gel and electrotransferred from the gel to a nitrocellulose membrane (Merck & Co., Inc., Whitehouse Station, NJ, USA). Following blocking with 5% skimmed milk in phosphate-buffered saline, the membranes were immunoblotted with the primary antibodies, namely, CDK6 (GeneTex, San Antonio, Texas, USA), DOT1L, H3, H3K79me2, cyclin D3, and GAPDH (Abcam, Cambridge, UK) at 4 °C overnight. Specifically bound HRP-conjugated secondary antibodies were detected using an ECL detection system (ChemiDocTM XRS+ machine, Bio-Rad Laboratories). Protein levels of GAPDH and H3 were employed as loading controls. Densitometric analyses were performed using ImageJ software. Relative quantification was carried out after normalization to the band intensities of GAPDH or H3. A Mann-Whitney test was performed to assess the difference of protein expression between groups. Each experiment was performed in triplicate and repeated at least 3 times.

### In vitro cell viability

Cell proliferation was evaluated using a colorimetric assay as described previously [26]. Cell viability was measured by a commercially Cell Counting Kit (CCK-8, Beyotime Institute of Biotechnology, China). The indicated cells were seeded into 96-well plates at a density of 10^4^ cells per well. The drugs were added when the plates were seeded. After incubation for 3, 6, 9, and 12 days, cell proliferation in 96-well plates was measured and 10 μL CCK-8 reagent was added to each well and incubated at 37 °C for 4 h. Absorbance was measured at 450 nm by using a spectrophotometer. Each experiment was performed in triplicate and repeated at least 3 times.

### Colony formation assay

The colony formation assay was performed on the indicated cells. For each group, 800 survived cells/well were incubated in 6-well plates, containing 2.5 ml complete medium per well, followed by incubation for 10 days. At the indicated time point, cells were washed twice with PBS, treated with crystal violet for 10 min, washed, then counted, and measured. All the experiments were performed at least 3 times. The colonies with >40 cells were subsequently counted under a phase contrast microscope at ×40 magnification.

#### Cell cycle and cell apoptosis analyses

Cell cycle and cell apoptosis analyses were determined by flow cytometry (BD, UA) [27, 28]. In this experiment, the SK-OV-3 and TOV21G cells were treated with 10 μM SGC0946 and EPZ004777 for 12 days, respectively. Then, the cells treated above were harvested and washed with phosphate-buffered saline (PBS). For cell cycle analysis, the pellets were fixed with ice-cold 70% ethanol overnight at 4 °C. Cell pellets were then suspended in 300 μl PBS containing 100 μg/ml RNase for 30 min at room temperature, and then 10 μg/ml propidium iodide (Sigma-Aldrich Co., St Louis, MO, USA). The distribution of cells in the different phases of the cell cycle was analyzed from DNA histograms using BD Cell Quest software (Becton Dickinson Biosciences, San Diego, CA, USA). For cell apoptosis analysis, cells were resuspended in 500 μl binding buffer (Annexin V-FITC/PI Apoptosis Detection Kit (BD Biosciences)), containing 5 μl of annexin V-fluorescein isothiocyanate (FITC) stock and 5 μl of 20 μg/ml PI for determination of phosphatidylserine (PS) exposure on the outer plasma membrane. After incubation for 10 min at room temperature in a light-protected area, the cells were quantified by flow cytometry. Viable cells were annexin V- and PI-negative.

#### ChIP-qPCR and ChIP-Seq

ChIP assays were performed following the protocols as previously described [13, 29, 30]. In brief, crosslinking was performed with 1% formaldehyde at room temperature for 10 min and the reaction was stopped by treatment with 0.125 M glycine. The cell pellets were resuspended in 200 ml of SDS lysis buffer and sonicated using a Bioruptor (Cosmo Bio Co. Ltd, Tokyo, Japan). The cell lysates were then immunoprecipitated with specific antibodies overnight at 4 °C and further incubated with salmon sperm DNA coupled to protein A-agarose (Millipore) for 2 h at 4 °C. The precipitates were washed, eluted, and then reverse-crosslinked with 20 ml 5 M NaCl by incubating at 65 °C overnight. DNA fragments were precipitated from the eluate and dissolved in ddH_2_O. Eluted DNA fragments were analyzed by qPCR, or subjected to sequencing using next-generation Solexa sequencing.

### Animal studies

Female NOD-SCID mice (4 weeks old) were purchased from Beijing HFK Bio-Technology Co. Ltd. (Beijing, China). The studies were approved by the Committee on Ethics of Animal Experiments of Tongji Medical College. The mice were maintained in the accredited animal facility of Tongji Medical College. Upon injection of SK-OV-3 cells transduced with CMV-Fluc-IRES-RFP lentiviral particles into the ovarian bursa bilaterally of female mice, we have developed an orthotopic ovarian tumor model. SGC0946 (10 mg/kg per mouse) was administered intraperitoneal twice a week for 6 weeks. The size of tumor was monitored in living mice by optical imaging of luciferase activity using the IVIS Spectrum system (Caliper, Xenogen, USA). After anaesthetization with 3% pentobarbital sodium, mice were imaged 10 min after intraperitoneal injection of 100 mg/kg D-luciferin. In the experimental metastasis model [31, 32], tumor cells were injected into mice via the tail vein (2 × 10^6^ cells/mouse). At each time option, mice were monitored using the IVIS SPECTRUM system according to the method above. Total flux (photons/s) was quantified using Living Image version 4.3.1 software.

### Statistical analysis

Chi-squared tests were used to evaluate whether DOT1L expression was correlated with clinicopathological factors of OC. Overall survival (OS) and progression-free survival (PFS) curves were plotted using the Kaplan-Meier analysis followed by two-sided log-rank tests. The univariate and multivariate Cox proportional hazards modeling was used to evaluate prognostic significance. For cell-based assays, differences between groups were assessed by two sided Student’s *t* tests, unless indicated otherwise. Data were analyzed using SPSS 18.0 statistic software (SPSS Inc., Chicago, IL). Each experiment was repeated at least 3 times with comparable results, unless indicated otherwise. Significance was assessed at the level of *P* < 0.05. (*P < 0.05, **P < 0.01, and ***P < 0.001, ****P < 0.0001).

## Results

### DOT1L and H3K79 methylation expression in normal ovarian tissues and benign, borderline, and malignant ovarian tumors

**Fig. 1:**
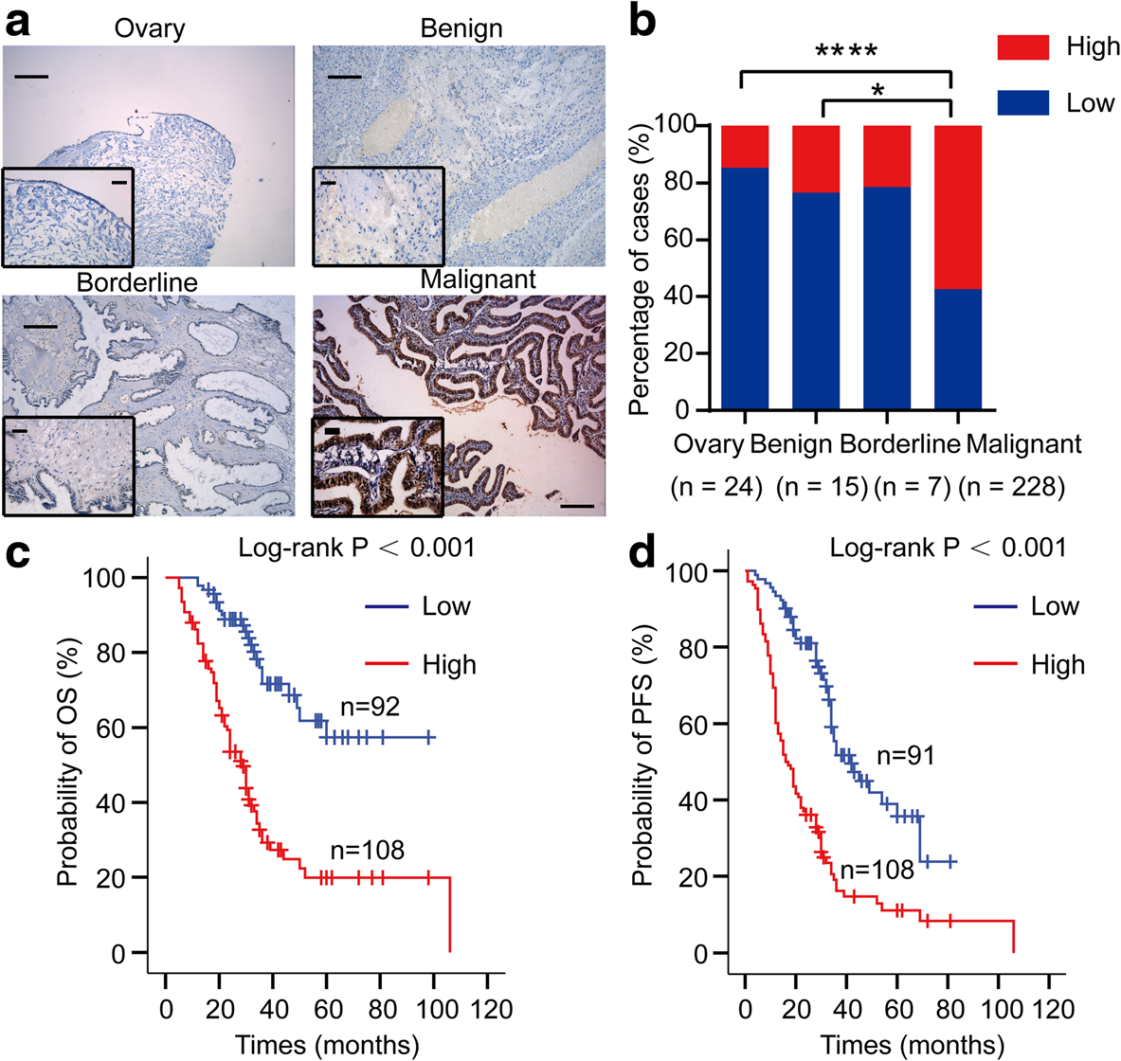
DOT1L protein expression in human ovarian tissues and Kaplan-Meier plots. **a** Immunohistochemical staining of DOT1L protein in human ovarian tissues. A *brown color* in cancer cells is considered as a positive staining. Negative control without first antibody is performed in the normal ovarian tissue. Representative images of DOT1L expression in normal ovarian tissues and benign, borderline, and malignant ovarian tumors are shown. Original magnification ×100 and ×400. *Scale bar* 100 and 25 μm, respectively. **b** The case rate of DOT1L high and low expression. For comparison between two groups, *χ* ^2^ test was applied. **c** Overall survival (OS) rate in patients with high DOT1L expression was significantly lower than in patients with no or low DOT1L expression. **d** Progression-free survival (PFS) rate in patients with high DOT1L expression was significantly lower than in patients with no or low DOT1L expression

Immunohistochemical staining for DOT1L (Fig. 1a) and H3K79 methylation (Additional file 2: Figure S1a) expression was performed on 24 normal ovarian tissues, 15 benign ovarian tumors, 7 borderline ovarian tumors, and 228 malignant ovarian tumors. At the time of analysis, 94 (45.6%) of the 206 evaluable patients had died and 131 (63.6%) of the 206 evaluable patients had experienced disease progression, resulting in a median OS of 36 months (95% CI, 24.5 to 47.5 months) and median PFS of 30 months (95% CI, 25.1 to 34.9 months). The average length of follow-up time for the 112 patients still alive was 38.5 months (range, 10 to 81 months). The percentage of high DOT1L expression was 14.6% in normal ovarian tissues, 23.4% in benign ovarian tumors, 21.5% in borderline ovarian tumors, and increased to 57.5% in malignant ovarian tumors (normal vs. malignant, *P* < 0.0001; benign vs. malignant, *P* = 0.012; borderline vs. malignant, *P* = 0.082; normal vs. benign, *P* = 0.69; normal vs. borderline, *P* = 0.62; benign vs. borderline, *P* = 1.00) (Fig. 1b). Similarly, H3K79 methylation was significantly increased in malignant ovarian cancer with 59.3% of high H3K79 methylation, while normal ovarian tissues with 16.65%, benign ovarian tumors with 16.65%, and borderline ovarian tumors with 37.75% (normal or benign vs. malignant, *P* < 0.0001; borderline vs. malignant, *P* = 0.0045; normal or benign vs. borderline, *P* = 0.0014) (Additional file 2: Figure S1b).

### Association between DOT1L expression and clinicopathological parameters of OC

To clarify the potential prognostic roles of DOT1L in OC development and progression, we evaluated the association of DOT1L expression with various clinical features of 235 cases of OC, including 7 borderline ovarian tumors and 228 malignant ovarian tumors (Table 1). High expression of DOT1L was associated with FIGO stage (*P* = 0.001), histologic grade (*P* = 0.012), and lymph node metastasis (*P* = 0.036). However, there was no significant association between DOT1L expression and patients’ age (*P* = 0.729), ascites (*P* = 0.944), and level of preoperative CA125 (*P* = 0.176) (Table 1).

**Table 1:** Association of DOT1L expression with the clinicopathological features of patients with borderline and malignant ovarian tumors

Characteristics	Sample	DOT1L expression		*χ* ^2^	*P*
		Low expression	High expression		
Age at diagnosis	235			0.120	0.729
≤50	114	51 (44.7%)	63 (55.3%)		
>50	117	55 (47.0%)	62 (53.0%)		
Unknown	4				
FIGO stage	235			17.655	0.001
I	20	18 (90.0%)	2 (10.0%)		
II	17	8 (47.1%)	9 (52.9%)		
III	159	66 (41.5%)	93 (58.5%)		
IV	29	11 (37.9%)	18 (62.1%)		
Unknown	10				
Histologic grade	235			8.831	0.012
Grade 1	40	27 (67.5%)	13 (32.5%)		
Grade 2	58	24 (41.4%)	34 (58.6%)		
Grade 3	120	50 (41.7%)	70 (58.3%)		
Unknown	17				
Ascites	235			0.005	0.944
Yes	173	78 (45.1%)	95 (54.9%)		
No	36	16 (44.4%)	20 (55.6%)		
Unknown	26				
LN metastasis	235			4.392	0.036
Yes	73	29 (39.7%)	44 (60.3%)		
No	106	59 (55.7%)	47 (44.3%)		
Unknown	56				
Preoperative CA125 (U/ml)	235			1.829	0.176
≤600	68	33 (48.5%)	35 (51.5%)		
>600	80	30 (37.5%)	50 (62.5%)		
Unknown	87				

### High expression of DOT1L as an independent prognostic marker of OC

We used only 228 malignant ovarian tumor samples for survival analysis. The Kaplan-Meier survival analysis indicated that OC patients with high expression of DOT1L exhibited a significantly reduced OS (*P* < 0.001, Fig. 1c) and PFS (*P* < 0.001, Fig. 1d) than those with low expression. In addition, we determined prognostic factors of OS and PFS using the univariate (Additional file 3: Table S2) and multivariate (Table 2) Cox regression model. The results identified high expression of DOT1L (*P* < 0.001), and age (*P* = 0.002) as prognostic factors of OS independent of other clinicopathological factors (Table 2). Meanwhile, the results identified high expression of DOT1L (*P* < 0.001), age (*P* = 0.005), and level of preoperative CA125 (*P* < 0.001) as prognostic factors of PFS independent of other clinicopathological factors (Table 2). Taken together, these results suggest that DOT1L expression was significantly increased in malignant ovarian tumors. Higher DOT1L expression was associated with advanced FIGO stage, poor histopathological grade, and lymph node metastasis of OC patients. Importantly, DOT1L expression was identified as an independent prognostic factor for OC.

**Table 2:** Multivariate Cox regression analysis of the clinicopathogical parameters for overall survival and progression free survival

	Overall survival			Progression free survival		
	HR	95% CI	*P*	HR	95% CI	*P*
FIGO stage (early stage vs. advanced stage)	1.127	0.357–3.562	0.838	1.744	0.576–5.288	0.325
Histologic grade (well vs. poor and moderate)	0.615	0.256–1.475	0.276	0.904	0.393–2.077	0.811
Ascites (no vs. yes)	0.850	0.381–1.897	0.692	0.667	0.337–1.323	0.247
LN metastasis (no vs. yes)	0.924	0.460–1.856	0.823	0.786	0.410–1.506	0.468
Age (≤50 vs. >50 years)	2.920	1.466–5.815	0.002	2.403	1.302–4.435	0.005
Preoperative CA125 (U/ml) (≤600 vs. >600)	0.548	0.280–1.073	0.079	0.327	0.177–0.607	0.000
DOT1L expression (low vs. high)	4.705	2.160–10.248	0.000	8.884	3.974–19.861	0.000

*HR* hazard ratio, *CI* confidence interval

### Knocking down of DOT1L inhibits the proliferation of ovarian cancer cells and induces G1 phase arrest

**Fig. 2:**
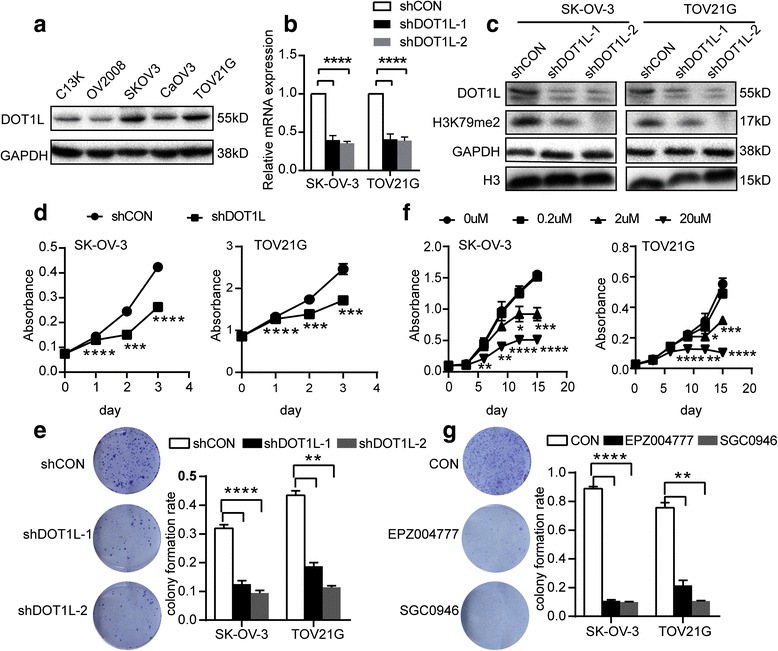
Knocking down of DOT1L inhibits the proliferation of ovarian cancer cells. **a** Western blot analysis of DOT1L expression in various ovarian cancer cell lines. GAPDH was used as loading control. **b** RT-PCR analysis of endogenous DOT1L mRNA expression in SK-OV-3 and TOV21G cells that were transfected with shRNAs lentiviral articles against DOT1L as indicated in the figure. The shRNAs were able to knockdown DOT1L. **c** Western blot analysis of SK-OV-3 and TOV21G cells transfected with shDOT1L shRNAs. The blot was probed with DOT1L and H3K79me2 antibodies. GAPDH and H3 were used as loading control. **d** The cell proliferation of SK-OV-3 and TOV21G was determined by CCK-8 assay after shDOT1L shRNAs transfection (*n* = 3). The results are representative of three independent experiments. *P* < 0.05. **e** The colony formation assay was performed in SK-OV-3 and TOV21G cells after shDOT1L shRNAs transfection (*n* = 3). Representative images (*left*) and the histogram (*right*) of three independent experiments were shown. *P* < 0.05. **f** SK-OV-3 and TOV21G cells were seeded in 96-well plates and cultured in the presence of SGC0946 at variable concentrations (0, 0.2, 2, or 20 μM) for up to 12 days. Then, the OD450 values of corresponding cells were measured at 0, 3, 6, 9, and 12 days after SGC0946 treatment. The results are representative of three independent experiments. (*n* = 3, *P* < 0.05). **g** The colony formation assay was performed in SK-OV-3 and TOV21G cells after 10 μM EPZ004777 and 10 μM SGC0946 treatment for up to 12 days. Representative images (*left*) and the histogram (*right*) of three independent experiments were shown. (*n* = 3, *P* < 0.05)

Given the fact that DOT1L has strong correlation with malignant behavior of ovarian cancer, such as late FIGO stage and poor histologic grade, a loss-of-function approach was applied to explore the function of DOT1L. First, we detected the expression levels of DOT1L in various OC cell lines by Western blot (Fig. 2a). SK-OV-3 and TOV21G cells have a relatively high expression of DOT1L compared with the other cell lines; therefore, we chose these two cell lines to knock down DOT1L. Successful knockdown by shDOT1L shRNA lentiviral particles was verified by RT-PCR (Fig. 2b) and Western blot (Fig. 2c). It was worth to note that the level of H3K79me2 was reduced responding to knockdown of DOT1L protein expression. To determine if knock-down of DOT1L expression by shRNA could lead to a decrease in the proliferation and survival of OC cells, CCK8 and colony formation assay were performed. The OD450 values of the SK-OV-3 and TOV21G cells transfected with shDOT1L shRNAs lentiviral particles showed significant decrease (all *P* < 0.05), compared with those cells in the control groups (Fig. 2d). The colony formations of transfected SK-OV-3 and TOV21G cells were also significantly reduced compared with the control groups (all *P* < 0.05, Fig. 2e). Consistent with knock-down of DOT1L expression genetically using shRNA, the proliferation and survival of OC cells were also reduced by inhibition of DOT1L enzymatic activity pharmacologically with EPZ004777 and SGC0946 treatment (Fig. 2f and g). SGC0946 was capable of inhibiting the growth of both SK-OV-3 and TOV21G cells in a dose- and time-dependent manner (Fig. 2f). Both EPZ004777 and SGC0946 could reduce the colony formation rate of SK-OV-3 and TOV21G cells (Fig. 2g). In order to evaluate the therapeutic effects of DOT1L inhibitors on a larger ovarian cancer cell line panel in vitro, we chose OV90 and CaOV3, which could represent high-grade serous ovarian cancer for CCK8 and colony formation assay [33]. Intriguingly, SGC0946 was capable of inhibiting the growth of both OV90 and CaOV3 cells in a dose- and time-dependent manner (Additional file 2: Figure S1c). Both EPZ004777 and SGC0946 could reduce the colony formation rate of OV90 and CaOV3 cells (Additional file 2: Figure S1d).

**Fig. 3:**
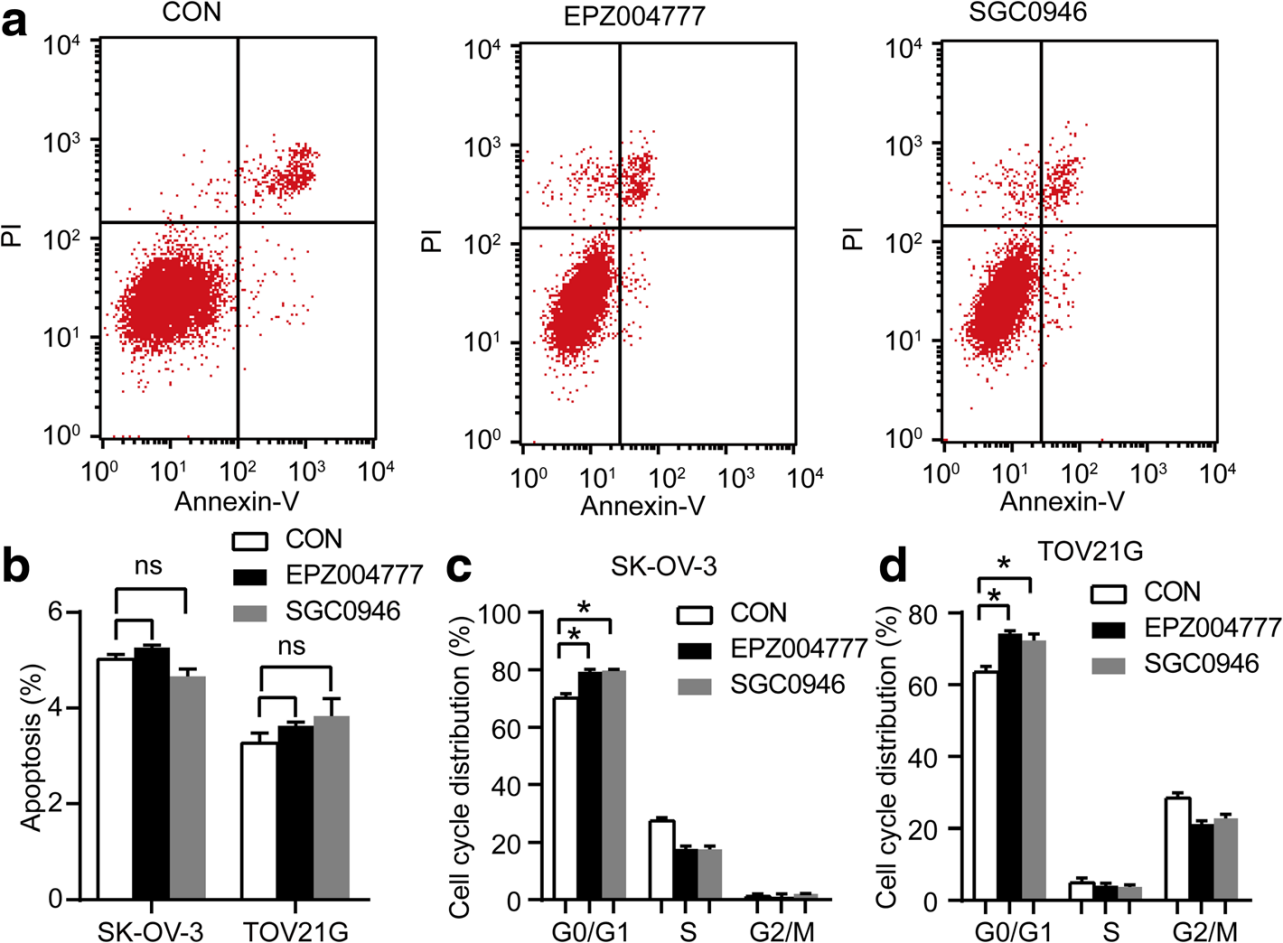
Effect of blocking DOT1L in the cell apoptosis and cell cycle. After exposure to 10 μM EPZ004777 and SGC0946 seperately for 12 days in both SK-OV-3 and TOV21G cells, cell apoptosis and cell cycle analyses in each group were determined by flow cytometry. **a** The typical images and **b** the proportion of apoptosis cell were measured in SK-OV-3 and TOV21G cells. The proportion of cell cycle distribution in SK-OV-3 cells (**c**) and in TOV21G cells (**d**) was shown. *Columns* are the mean of triplicate samples

To further determine the reason that knockdown DOT1L inhibits ovarian cancer cell proliferation, we next performed flow cytometric analysis of apoptosis and cell cycle. None effect of DOT1L on apoptosis of either SK-OV-3 or TOV21G cells was seen after treatment with EPZ004777 and SGC0946 for up to 12 days (Fig. 3a, b). However, EPZ004777 and SGC0946 treatment induced increased G1 population and decreased S phase and G2/M phase cells in asynchronized SK-OV-3 and TOV21G cells (Fig. 3c, d, Additional file 4: Figure S2). Similar result was seen in both SK-OV-3 and TOV21G cells transfected with DOT1L shRNA lentivirus (GeneChem) (Additional file 5: Figure S3). These results demonstrate that blocking DOT1L in ovarian cancer could result in G1 arrest and thereby impede the cell proliferation in vitro*.*

### DOT1L regulates the transcription of G1 arrest genes CDK6 and CCND3 through H3K79 dimethylation

**Fig. 4:**
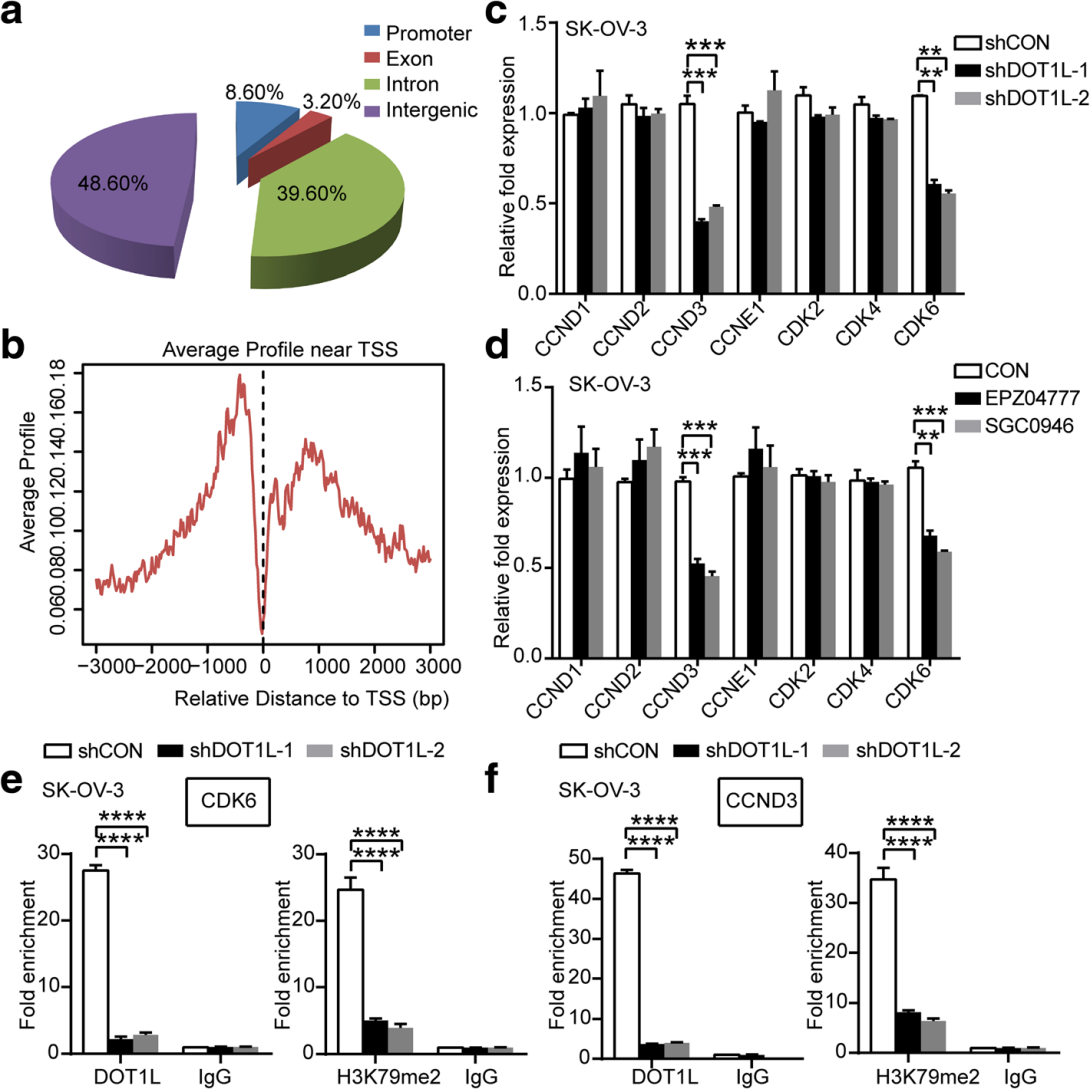
DOT1L regulates the transcription of G1 arrest genes CDK6 and CCND3 through H3K79 dimethylation. **a** Genomic distribution of DOT1L ChIP-seq peaks in SK-OV-3 cells. The proportion of DOT1L peaks was 8.60% distributed in promoters, 3.20% in exons, 39.60% in introns, and 48.6% in intergenics. **b** The average distribution of DOT1L-bound peaks near TSS. **c** and **d** RT-PCR detecting the change of primary G1 arrest genes, D-type cyclins (D1, D2, and D3), cyclin E, Cdks (2, 4, and 6) in mRNA levels in SK-OV-3 cells knocking down of DOT1L with shDOT1L shRNAs lentiviral particles (**c**) and DOT1L inhibitors, EPZ004777 and SGC0946 (**d**) (*P* < 0.05, Student’s *t* test, data represented as mean±SD). **e** and **f** DOT1L transcriptionally regulates the expression of CDK6 (**e**) and CCND3 (**f**) through induction of H3K79 dimethylation in the binding regions of these genes. Chromatin immunoprecipitation (ChIP) PCR assays were performed in a loss-of-function system. SK-OV-3 cells were transfected with shDOT1L shRNAs and shCON lentiviral particles and ChIP assays using anti-DOT1L and anti-H3K79me2 antibodies were performed with ChIP primers targeting the binding regions of CDK6 and CCND3 (*P* < 0.05, Student’s *t* test, data represented as mean±SD)

**Fig. 5:**
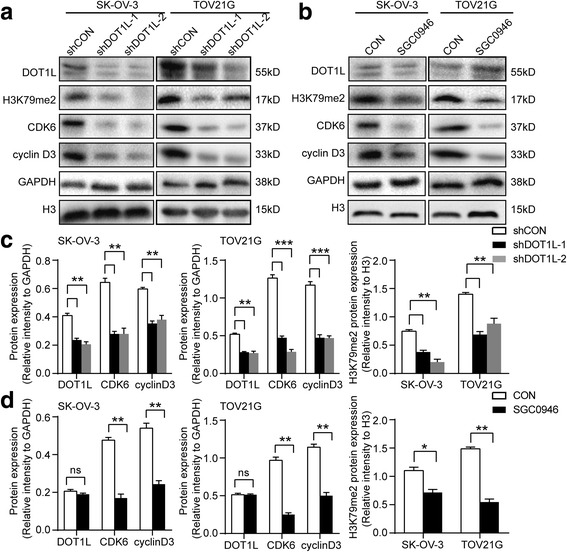
Knocking down of DOT1L decreases the expression of CDK6 and cyclin D3 in ovarian cancer cells. Western blotting confirmed decrease of corresponding G1 arrest proteins, CDK6, and cyclin D3 protein levels in SK-OV-3 cells and TOV21G cells knocking down of DOT1L with shDOT1L shRNAs (**a**) and DOT1L inhibitor, SGC0946 (**b**). **c** and **d** Densitometric analysis of the Western blot. Data shown are mean±SD and a two-tailed Mann-Whitney test was used for statistical analysis (*P* < 0.05). GAPDH or H3 was loading as control

Given that knocking down of DOT1L could inhibit the proliferation of ovarian cancer cells in vitro and induce G1 arrest as mentioned above, we then proceeded to elucidate the molecular mechanism underlying this phenomenon. To identify genes regulated by DOT1L in ovarian cancer cells, we performed chromatin immune-precipitation coupled with high-throughput sequencing (ChIP-seq) analysis using ChIP-grade DOT1L antibody in SK-OV-3 cells. The proportion of DOT1L peaks was 8.60% distributed in promoters, 3.20% in exons, 39.60% in introns, and 48.6% in intergenics (Fig. 4a). The average distribution of DOT1L-bound peaks near TSS was shown in Fig. 4b. We then examined the change of expression in mRNA level of primary genes involved in G1 arrest responding to knocking down of DOT1L in OC cells. These primary G1 arrest genes include D-type cyclins (D1, D2, and D3), cyclin E, and Cdks (2, 4, and 6) [29, 30]. Downregulation of CCND3 and CDK6 were seen in SK-OV-3 cells and TOV21G cells both by DOT1L shRNAs silicing and treated with DOT1L inhibitors (Fig. 4c, d, Additional file 6: Figure S4a and b). However, we could not detect the change of other G1 arrest genes. Interestingly, among these downregulated genes responding to DOT1L-knockdown, we found the intron of CDK6 and CCND3 genes were bound to DOT1L, suggesting that CDK6 and CCND3 genes were the direct target genes of DOT1L (Additional file 7). To verify whether DOT1L directly regulated the transcription of CDK6 and CCND3 through the H3K79 dimethylation, we performed ChIP-PCR using ChIP-grade antibodies for DOT1L and H3K79me2, targeting the detected binding sites nearby with three different primer sets. In accordance with the results above, CDK6 levels in DOT1L-immunoprecipitants were decreased by more than 12 times in the shDOT1L transfected SK-OV-3 cells compared with the control (*P* < 0.0001, Student’s *t* test), and an approximately 5-fold decrease of CDK6 was observed in the H3K79me2-enriched cell lysates (*P* < 0.0001, Student’s *t* test) (Fig. 4e). Likewise, we observed a decrease in the CCND3 levels by near 13 times in the DOT1L-immunoprecipitants of the lysates from shDOT1L transfected SK-OV-3 cells (*P* < 0.0001, Student’s *t* test), and by more than 4 times decrease in the H3K79me2-immunoprecipitants (*P* < 0.0001, Student’s *t* test) (Fig. 4f).Similar results were seen in TOV21G cells (Additional file 6: Figure S4). We further confirmed downregulation of CDK6 and cyclin D3 in both SK-OV-3 and TOV21G cells knockdown of DOT1L at the protein level, which are critical for normal G1 phase progression [34, 35] (Fig. 5). These results support that blocking DOT1L can result in G1 arrest through its H3K79 dimethylation activity.

### Effect of inhibition of DOT1L pharmacologically on tumor progression in vivo

**Fig. 6:**
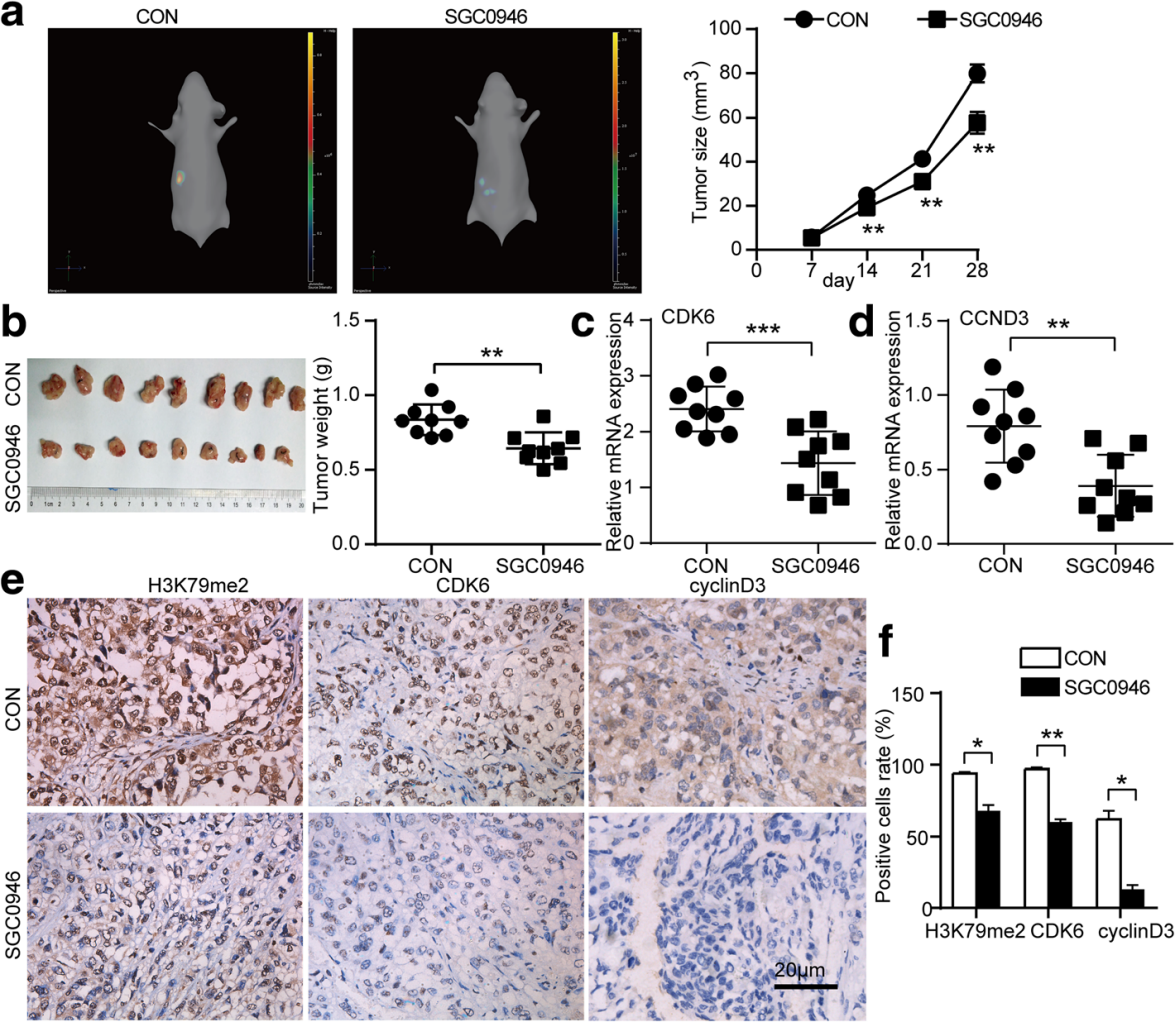
Effect of inhibition of DOT1L pharmacologically on tumor progression in vivo. After orthotopic transplantation of tumors performed by SK-OV-3 cells transduced with CMV-Fluc-IRES-RFP lentiviral particles, BALB/c-nude mice were treated with SGC0946 (10 mg/kg body weight) or vehicle control twice a week for 6 weeks by intraperitoneal injection. The size of tumor was closely monitored by three-dimensional reconstruction of in vivo bioluminescence images. **a** The representative images (*left*) were shown and tumor size (*right*) was calculated (*n* = 9 per group, *P* < 0.05, Student’s *t* test, data represented as mean±SD). **b** The mice were sacrificed after being monitored using the IVIS SPECTRUM system. Photograph of tumor masses (*left*) was shown and the tumor weight (*right*) was measured (*n* = 9 per group, *P* < 0.05, Student’s *t* test, data represented as mean±SD). RT-PCR was performed on the tumor masses from the mice mentioned above to analyze the expression of CDK6 (**c**) and CCND3 (**d**). GAPDH was used as a loading control. **e** Representative IHC images for H3K79me2, CDK6, and cyclin D3 in the mice were shown. **f** The positive cells of H3K79me2, CDK6, and cyclin D3 were measured

To determine whether DOT1L has a functional role in tumor progression in vivo, we used a mouse orthotopic xenograft ovarian cancer model. Mice treated with SGC0946 displayed obviously slower progress in tumor growth compared with the mice untreated (Fig. 6a). After the sacrifice of these mice, tumor masses were removed and weighted. The size and weight of tumor masses treated with SGC0946 were significantly smaller than the untreated group (Fig. 6b). We performed RT-PCR analysis for CDK6 and CCND3 on these tumor masses. CDK6, as well as CCND3, was significantly decreased in SGC0946-treated group than the untreated group (Fig. 6c, d). Representative IHC images for H3K79me2, CDK6, and cyclin D3 in the mice were shown to confirm the inhibition of DOT1L enzymatic activity upon SGC0946 treatment, showing impeded H3K79me2, CDK6, and cyclin D3 levels in the tumors (Fig. 6e, f). Taken together, these results demonstrated that DOT1L inhibitors could inhibit tumor growth in vivo.

## Discussion

This is the first study to examine DOT1L expression and H3K79 methylation in ovarian cancer. In human, DOT1L is involved in the oncogenesis of several leukemia subtypes, mostly characterized by chromosomal translocations involving the mixed lineage leukemia (MLL) gene [36]. To identify the role of DOT1L in ovarian cancer, we performed immunohistochemistry and found that DOT1L expression was significantly increased in malignant ovarian cancer. In addition, H3K79 methylation was also significantly increased in malignant ovarian cancer, indicating the correlation of DOT1L and H3K79 methylation in ovarian cancer. Higher DOT1L expression was associated with advanced FIGO stage, poor histopathological grade, and lymph node metastasis of OC patients. Importantly, DOT1L expression was identified as an independent prognostic factor for OC, which raised the possibility that DOT1L might be correlated with malignant behavior of ovarian cancer. As expected, our in vitro experiment demonstrated that blocking DOT1L in ovarian cancer could lead to the decrease of ovarian cancer cell viability and colony-forming ability.

DOT1L has a critical role in regulating gene transcription, development, cell cycle progression, somatic reprogramming, and DNA damage repair [37–39]. Consistent with previous finding that DOT1L siRNA-transfected human lung cancer cells undergo G1 cell cycle arrest [40], we showed here that knocking down of DOT1L in ovarian cancer cells could result in G1 arrest and therefore impede the cell proliferation. It is worth mentioning that asynchronized cells were used for cell cycle analysis in this paper, due to the reason that DOT1L might be related with DNA replication [38, 39]. Most importantly, the present study provides mechanistic insight into the inhibition of ovarian cancer cell proliferation by blocking DOT1L via downregulation of CDK6 and CCND3 through H3K79 dimethylation, providing a novel mechanism of epigenetic regulation of DOT1L-mediated transcription of cell cycle genes. The D-type cyclins (cyclins D1, D2, and D3) are the first cyclins to be induced as cells enter the G1 phase of the cell cycle [34, 41, 42]. D-type cyclins associate with and activate cyclin-dependent kinases (CDK), such as CDK4 and CDK6 [34]. Specially, cyclin D3 was identified as a direct target of Notch1 that promotes cell-cycle progression and proliferation both in peripheral and leukemic T cells [43]. The data presented in this report demonstrated that DOT1L regulation of cell cycle was through CDK6 and CCND3. Although several studies reported that some histone modifications transcriptionally regulated the expression of cell cycle regulators such as CDK inhibitors to govern cell cycle progression [40, 44, 45], in fact, our data together with the described molecular functions identified CDK6 and cyclin D3 as transcriptionally regulated genes by DOT1L. Given the fact that the cell-cycle arrest network is complex, because the regulators involved act redundantly, have multiple substrates, and provide feedback control [46], the phenomenon was explainable.

The dynamic interplays among different chromatin post-transcription modifications control gene expression and provide novel opportunities for targeted combination therapies in multiple cancer types. Molecular targeting of specific histone methyltransferase is emerging as a new direction for cancer therapy [47]. Of the known histone methyltransferase enzymes, DOT1L is a promising target due to it being the only known H3K79 histone methyltransferase, its unique non-SET catalytic domain, and its role in promoting and maintaining MLL leukemogenesis [48, 49]. It is interesting to note that small molecule DOT1L inhibitors have been recently developed, and one of the DOT1L inhibitors is already under investigation in a phase I clinical trial in patients with MLL fusion gene-driven leukemia [37]. Fortunately, our data showed that inhibition of DOT1L enzymatic activity with EPZ004777 and SGC0946 treatment could suppress ovarian cancer cell proliferation in vitro, and furthermore, administration of SGC0946 in vivo resulted in significant inhibition of tumor progression in the ovarian orthotopic xenograft mice model. Consequently, DOT1L could have strong prospects for therapeutic target in ovarian cancer.

## Conclusions

Our findings first demonstrated that OC patients expressing high level of DOT1L have poorer OS and PFS compared with those with low DOT1L expression, and DOT1L expression can be regarded as an independent prognostic factor. Furthermore, DOT1L regulates the transcription of G1 phase genes CDK6 and CCND3 through H3K79 dimethylation, thereby knocking down of DOT1L could result in G1 arrest and therefore impede the cell proliferation and tumor progression both in vitro and in vivo. Our results suggest that DOT1L might be potential target for prognostic assessment and therapeutic intervention in ovarian cancer.

## Additional files

Additional file 1: Table S1. The primer sequences used for PCR. (DOCX 15 kb)
Additional file 2: Figure S1. Immunostaining of H3K79 methylation in human ovarian cancer specimens and effects of DOT1L inhibitors in vitro*.* (a) Immunohistochemical staining of H3K79 methylation in human ovarian tissues. A brown color in cancer cells is considered as a positive staining. Negative control without first antibody is performed in the normal ovarian tissue. Representative images of H3K79 methylation in normal ovarian tissues and benign, borderline, and malignant ovarian tumors are shown. Original magnification ×100 and ×400. Scale bar 100 μm and 25 μm, respectively. (b) The case rate of H3K79 methylation high and low expression. For comparison between two groups, *χ* ^2^ test was applied. (c) OV90 and CaOV3 cells were seeded in 96-well plates and cultured in the presence of SGC0946 at variable concentrations (0, 0.2, 2, or 20 μM) for up to 12 days. Then the OD450 values of corresponding cells were measured at 0, 3, 6, 9, and 12 days after SGC0946 treatment. The results are representative of three independent experiments. (*n* = 3, *P* < 0.05). (d) The colony formation assay was performed in OV90 and CaOV3 cells after 10 μM EPZ004777 and 10 μM SGC0946 treatment for up to 12 days. The histogram of three independent experiments was shown. (*n* = 3, *P* < 0.05). (TIF 5990 kb)
Additional file 3: Table S2. Univariate Cox regression analysis of the clinicopathogical parameters for overall survival and progression free survival. (DOCX 17 kb)
Additional file 4: Figure S2. Effect of blocking DOT1L in cell cycle. After exposure to 10 μM EPZ004777 and SGC0946 seperately for 12 days in both SK-OV-3 and TOV21G cells, cell apoptosis and cell cycle analyses in each group were determined by flow cytometry. The typical images of cell cycle in SK-OV-3 cells (a) and in TOV21G cells (b) were shown. (TIF 713 kb)
Additional file 5: Figure S3. Effect of blocking DOT1L using shRNAs in the cell apoptosis and cell cycle. ShDOT1L-1 and shDOT1L-2 shRNA lentiviral particles (GeneChem) were used to knock-down DOT1L expression in both SK-OV-3 and TOV21G cells, cell apoptosis and cell cycle analyses in each group were determined by flow cytometry. (a) The typical images and (b) the proportion of apoptosis cell were measured in SK-OV-3 and TOV21G cells. The typical images (c) and the proportion of cell cycle distribution (d) in SK-OV-3 cells were shown. The typical images (e) and the proportion of cell cycle distribution (f) in TOV21G cells were shown. Columns are the mean of triplicate samples. (TIF 1351 kb)
Additional file 6: Figure S4. DOT1L regulates the transcription of G1 arrest genes CDK6 and CCND3 through H3K79 dimethylation. (a) and (b) RT-PCR detecting the change of primary G1 arrest genes, D-type cyclins (D1, D2, and D3), cyclin E, Cdks (2, 4, and 6) in mRNA levels in TOV21G cells knocking down of DOT1L with shDOT1L shRNAs lentiviral particles (a) and DOT1L inhibitors, EPZ004777 and SGC0946 (b) (*P* < 0.05, Student’s *t* test, data represented as mean±SD). (c) and (d) DOT1L transcriptionally regulates the expression of CDK6 (c) and CCND3 (d) through induction of H3K79 dimethylation in the binding regions of these genes. Chromatin immunoprecipitation (ChIP) PCR assays were performed in a loss-of-function system. TOV21G cells were transfected with shDOT1L shRNAs and shCON lentiviral particles, and ChIP assays using anti-DOT1L and anti-H3K79me2 antibodies were performed with ChIP primers targeting the binding regions of CDK6 and CCND3 (*P* < 0.05, Student’s *t* test, data represented as mean±SD). (TIF 815 kb)
Additional file 7: Possible target genes of DOT1L analyzed from DOT1L ChIP-seq. (XLSX 854 kb)

## References

[1] Perren TJ, Swart AM, Pfisterer J, Ledermann JA, Pujade-Lauraine E, Kristensen G, Carey MS, Beale P, Cervantes A, Kurzeder C, du Bois A, Sehouli J, Kimmig R, Stahle A, Collinson F, Essapen S (2011). A phase 3 trial of bevacizumab in ovarian cancer. N Engl J Med.

[2] Zhang L, Han J, Jackson AL, Clark LN, Kilgore J, Guo H, Livingston N, Batchelor K, Yin Y, Gilliam TP, Gehrig PA, Sheng X, Zhou C, Bae-Jump VL (2016). NT1014, a novel biguanide, inhibits ovarian cancer growth in vitro and in vivo. J Hematol Oncol.

[3] Wang D, Zhu H, Ye Q, Wang C, Xu Y (2016). Prognostic value of KIF2A and HER2-Neu overexpression in patients with epithelial ovarian cancer. Medicine.

[4] Song Y, Wu F, Wu J (2016). Targeting histone methylation for cancer therapy: enzymes, inhibitors, biological activity and perspectives. J Hematol Oncol.

[5] Jackson RA, Chen ES (2016). Synthetic lethal approaches for assessing combinatorial efficacy of chemotherapeutic drugs. Pharmacol Ther.

[6] Nguyen AT, Zhang Y (2011). The diverse functions of Dot1 and H3K79 methylation. Genes Dev.

[7] Wakeman TP, Wang Q, Feng J, Wang XF (2012). Bat3 facilitates H3K79 dimethylation by DOT1L and promotes DNA damage-induced 53BP1 foci at G1/G2 cell-cycle phases. EMBO J.

[8] Chang MJ, Wu H, Achille NJ, Reisenauer MR, Chou CW, Zeleznik-Le NJ, Hemenway CS, Zhang W (2010). Histone H3 lysine 79 methyltransferase Dot1 is required for immortalization by MLL oncogenes. Cancer Res.

[9] Wang E, Kawaoka S, Yu M, Shi J, Ni T, Yang W, Zhu J, Roeder RG, Vakoc CR (2013). Histone H2B ubiquitin ligase RNF20 is required for MLL-rearranged leukemia. Proc Natl Acad Sci U S A.

[10] Bernt KM, Zhu N, Sinha AU, Vempati S, Faber J, Krivtsov AV, Feng Z, Punt N, Daigle A, Bullinger L, Pollock RM, Richon VM, Kung AL, Armstrong SA (2011). MLL-rearranged leukemia is dependent on aberrant H3K79 methylation by DOT1L. Cancer Cell.

[11] Gutierrez SE, Romero-Oliva FA (2013). Epigenetic changes: a common theme in acute myelogenous leukemogenesis. J Hematol Oncol.

[12] Xu J, Zhang W, Yan XJ, Lin XQ, Li W, Mi JQ, Li JM, Zhu J, Chen Z, Chen SJ (2016). DNMT3A mutation leads to leukemic extramedullary infiltration mediated by TWIST1. J Hematol Oncol.

[13] Cho MH, Park JH, Choi HJ, Park MK, Won HY, Park YJ, Lee CH, Oh SH, Song YS, Kim HS, Oh YH, Lee JY, Kong G (2015). DOT1L cooperates with the c-Myc-p300 complex to epigenetically derepress CDH1 transcription factors in breast cancer progression. Nat Commun.

[14] Oktyabri D, Ishimura A, Tange S, Terashima M, Suzuki T (2016). DOT1L histone methyltransferase regulates the expression of BCAT1 and is involved in sphere formation and cell migration of breast cancer cell lines. Biochimie.

[15] Singh V, Singh LC, Singh AP, Sharma J, Borthakur BB, Debnath A, Rai AK, Phukan RK, Mahanta J, Kataki AC, Kapur S, Saxena S (2015). Status of epigenetic chromatin modification enzymes and esophageal squamous cell carcinoma risk in northeast Indian population. Am J Cancer Res.

[16] Kryczek I, Lin Y, Nagarsheth N, Peng D, Zhao L, Zhao E, Vatan L, Szeliga W, Dou Y, Owens S, Zgodzinski W, Majewski M, Wallner G, Fang J, Huang E, Zou W (2014). IL-22(+)CD4(+) T cells promote colorectal cancer stemness via STAT3 transcription factor activation and induction of the methyltransferase DOT1L. Immunity.

[17] Pestell RG, Yu Z (2014). Long and noncoding RNAs (lnc-RNAs) determine androgen receptor dependent gene expression in prostate cancer growth in vivo. Asian J Androl.

[18] Annala M, Kivinummi K, Leinonen K, Tuominen J, Zhang W, Visakorpi T, Nykter M (2014). DOT1L-HES6 fusion drives androgen independent growth in prostate cancer. EMBO Mol Med.

[19] Donner I, Kiviluoto T, Ristimaki A, Aaltonen LA, Vahteristo P (2015). Exome sequencing reveals three novel candidate predisposition genes for diffuse gastric cancer. Familial Cancer.

[20] Zhang L, Deng L, Chen F, Yao Y, Wu B, Wei L, Mo Q, Song Y (2014). Inhibition of histone H3K79 methylation selectively inhibits proliferation, self-renewal and metastatic potential of breast cancer. Oncotarget.

[21] Liu D, Zhang XX, Xi BX, Wan DY, Li L, Zhou J, Wang W, Ma D, Wang H, Gao QL (2014). Sine oculis homeobox homolog 1 promotes DNA replication and cell proliferation in cervical cancer. Int J Oncol.

[22] Vos MC, van der Wurff AA, Bulten J, Kruitwagen R, Feijen H, van Kuppevelt TH, Hendriks T, Massuger LF (2016). Limited independent prognostic value of MMP-14 and MMP-2 expression in ovarian cancer. Diagn Pathol.

[23] Kamat AA, Fletcher M, Gruman LM, Mueller P, Lopez A, Landen CN, Han L, Gershenson DM, Sood AK (2006). The clinical relevance of stromal matrix metalloproteinase expression in ovarian cancer. Clin Cancer Res.

[24] Wei JC, Yang J, Liu D, Wu MF, Qiao L, Wang JN, Ma QF, Zeng Z, Ye SM, Guo ES, Jiang XF, You LY, Chen Y, Zhou L, Huang XY, Zhu T (2017). Tumor-associated lymphatic endothelial cells promote lymphatic metastasis by highly expressing and secreting SEMA4C. Clin Cancer Res.

[25] Tian Y, Wu K, Liu Q, Han N, Zhang L, Chu Q, Chen Y (2016). Modification of platinum sensitivity by KEAP1/NRF2 signals in non-small cell lung cancer. J Hematol Oncol.

[26] Ji T, Gong D, Han Z, Wei X, Yan Y, Ye F, Ding W, Wang J, Xia X, Li F, Hu W, Lu Y, Wang S, Zhou J, Ma D, Gao Q (2013). Abrogation of constitutive Stat3 activity circumvents cisplatin resistant ovarian cancer. Cancer Lett.

[27] Selvakumar E, Hsieh TC (2008). Regulation of cell cycle transition and induction of apoptosis in HL-60 leukemia cells by lipoic acid: role in cancer prevention and therapy. J Hematol Oncol.

[28] Feng Z, Yao Y, Zhou C, Chen F, Wu F, Wei L, Liu W, Dong S, Redell M, Mo Q, Song Y (2016). Pharmacological inhibition of LSD1 for the treatment of MLL-rearranged leukemia. J Hematol Oncol.

[29] Truax AD, Greer SF (2012). ChIP and Re-ChIP assays: investigating interactions between regulatory proteins, histone modifications, and the DNA sequences to which they bind. Methods Mol Biol.

[30] Liu D, Li L, Zhang XX, Wan DY, Xi BX, Hu Z, Ding WC, Zhu D, Wang XL, Wang W, Feng ZH, Wang H, Ma D, Gao QL (2014). SIX1 promotes tumor lymphangiogenesis by coordinating TGFbeta signals that increase expression of VEGF-C. Cancer Res.

[31] Liu D, Zhang XX, Wan DY, Xi BX, Ma D, Wang H, Gao QL (2014). Sine oculis homeobox homolog 1 promotes alpha5beta1-mediated invasive migration and metastasis of cervical cancer cells. Biochem Biophys Res Commun.

[32] Dong R, Qiang W, Guo H, Xu X, Kim JJ, Mazar A, Kong B, Wei JJ (2016). Histologic and molecular analysis of patient derived xenografts of high-grade serous ovarian carcinoma. J Hematol Oncol.

[33] Beaufort CM, Helmijr JC, Piskorz AM, Hoogstraat M, Ruigrok-Ritstier K, Besselink N, Murtaza M, van IJcken WF, Heine AA, Smid M, Koudijs MJ, Brenton JD, Berns EM, Helleman J (2014). Ovarian cancer cell line panel (OCCP): clinical importance of in vitro morphological subtypes. PLoS One.

[34] Murray AW (2004). Recycling the cell cycle: cyclins revisited. Cell.

[35] Hannon GJ, Beach D (1994). p15INK4B is a potential effector of TGF-beta-induced cell cycle arrest. Nature.

[36] Daigle SR, Olhava EJ, Therkelsen CA, Basavapathruni A, Jin L, Boriack-Sjodin PA, Allain CJ, Klaus CR, Raimondi A, Scott MP, Waters NJ, Chesworth R, Moyer MP, Copeland RA, Richon VM, Pollock RM (2013). Potent inhibition of DOT1L as treatment of MLL-fusion leukemia. Blood.

[37] Wong M, Polly P, Liu T (2015). The histone methyltransferase DOT1L: regulatory functions and a cancer therapy target. Am J Cancer Res.

[38] Fu H, Maunakea AK, Martin MM, Huang L, Zhang Y, Ryan M, Kim R, Lin CM, Zhao K, Aladjem MI (2013). Methylation of histone H3 on lysine 79 associates with a group of replication origins and helps limit DNA replication once per cell cycle. PLoS Genet.

[39] Oksenych V, Zhovmer A, Ziani S, Mari PO, Eberova J, Nardo T, Stefanini M, Giglia-Mari G, Egly JM, Coin F (2013). Histone methyltransferase DOT1L drives recovery of gene expression after a genotoxic attack. PLoS Genet.

[40] Kim W, Kim R, Park G, Park JW, Kim JE (2012). Deficiency of H3K79 histone methyltransferase Dot1-like protein (DOT1L) inhibits cell proliferation. J Biol Chem.

[41] Dyson N (1998). The regulation of E2F by pRB-family proteins. Genes Dev.

[42] Sherr CJ, Roberts JM (2004). Living with or without cyclins and cyclin-dependent kinases. Genes Dev.

[43] Joshi I, Minter LM, Telfer J, Demarest RM, Capobianco AJ, Aster JC, Sicinski P, Fauq A, Golde TE, Osborne BA (2009). Notch signaling mediates G1/S cell-cycle progression in T cells via cyclin D3 and its dependent kinases. Blood.

[44] Barradas M, Anderton E, Acosta JC, Li S, Banito A, Rodriguez-Niedenfuhr M, Maertens G, Banck M, Zhou MM, Walsh MJ, Peters G, Gil J (2009). Histone demethylase JMJD3 contributes to epigenetic control of INK4a/ARF by oncogenic RAS. Genes Dev.

[45] He J, Kallin EM, Tsukada Y, Zhang Y (2008). The H3K36 demethylase Jhdm1b/Kdm2b regulates cell proliferation and senescence through p15(Ink4b). Nat Struct Mol Biol.

[46] Ruijtenberg S, van den Heuvel S (2015). G1/S inhibitors and the SWI/SNF complex control cell-cycle exit during muscle differentiation. Cell.

[47] Kim W, Choi M, Kim JE (2014). The histone methyltransferase Dot1/DOT1L as a critical regulator of the cell cycle. Cell Cycle.

[48] Min J, Feng Q, Li Z, Zhang Y, Xu RM (2003). Structure of the catalytic domain of human DOT1L, a non-SET domain nucleosomal histone methyltransferase. Cell.

[49] Feng Q, Wang H, Ng HH, Erdjument-Bromage H, Tempst P, Struhl K, Zhang Y (2002). Methylation of H3-lysine 79 is mediated by a new family of HMTases without a SET domain. Curr Biol.

