# Supplementary material for: Prognostic and therapeutic value of disruptor of telomeric silencing-1-like (DOT1L) expression in patients with ovarian cancer
Source: J Hematol Oncol. 2017 Jan 23;10:29. doi: 10.1186/s13045-017-0400-8 (PMC5259947; doi:10.1186/s13045-017-0400-8)
Supplement: Additional file 1: Table S1. — The primer sequences used for PCR. (DOCX 15 kb) [file 13045_2017_400_MOESM1_ESM.docx]

**Additional file 1: Table S1**

**The primer sequences used for PCR**

| Primer name | Primer sequence (Forward) | Primer sequence (Reverse) |
| --- | --- | --- |
| GAPDH | CTGGGCTACACTGAGCACC | AAGTGGTCGTTGAGGGCAATG |
| DOT1L | GAGACCTCCTTCGACCTGGT | CGACGCCATAGTGATGTTTGC |
| CCND1 | CAATGACCCCGCACGATTTC | CATGGAGGGCGGATTGGAA |
| CCND2 | TTTGCCATGTACCCACCGTC | AGGGCATCACAAGTGAGCG |
| CCND3 | TACCCGCCATCCATGATCG | AGGCAGTCCACTTCAGTGC |
| CCNE1 | AAGGAGCGGGACACCATGA | ACGGTCACGTTTGCCTTCC |
| CDK2 | CCAGGAGTTACTTCTATGCCTGA | TTCATCCAGGGGAGGTACAAC |
| CDK4 | TCAGCACAGTTCGTGAGGTG | GTCCATCAGCCGGACAACAT |
| CDK6 | GCTGACCAGCAGTACGAATG | GCACACATCAAACAACCTGACC |
