# Supplementary figures and images for: Prognostic and therapeutic value of disruptor of telomeric silencing-1-like (DOT1L) expression in patients with ovarian cancer
Source: J Hematol Oncol. 2017 Jan 23;10:29. doi: 10.1186/s13045-017-0400-8 (PMC5259947; doi:10.1186/s13045-017-0400-8)

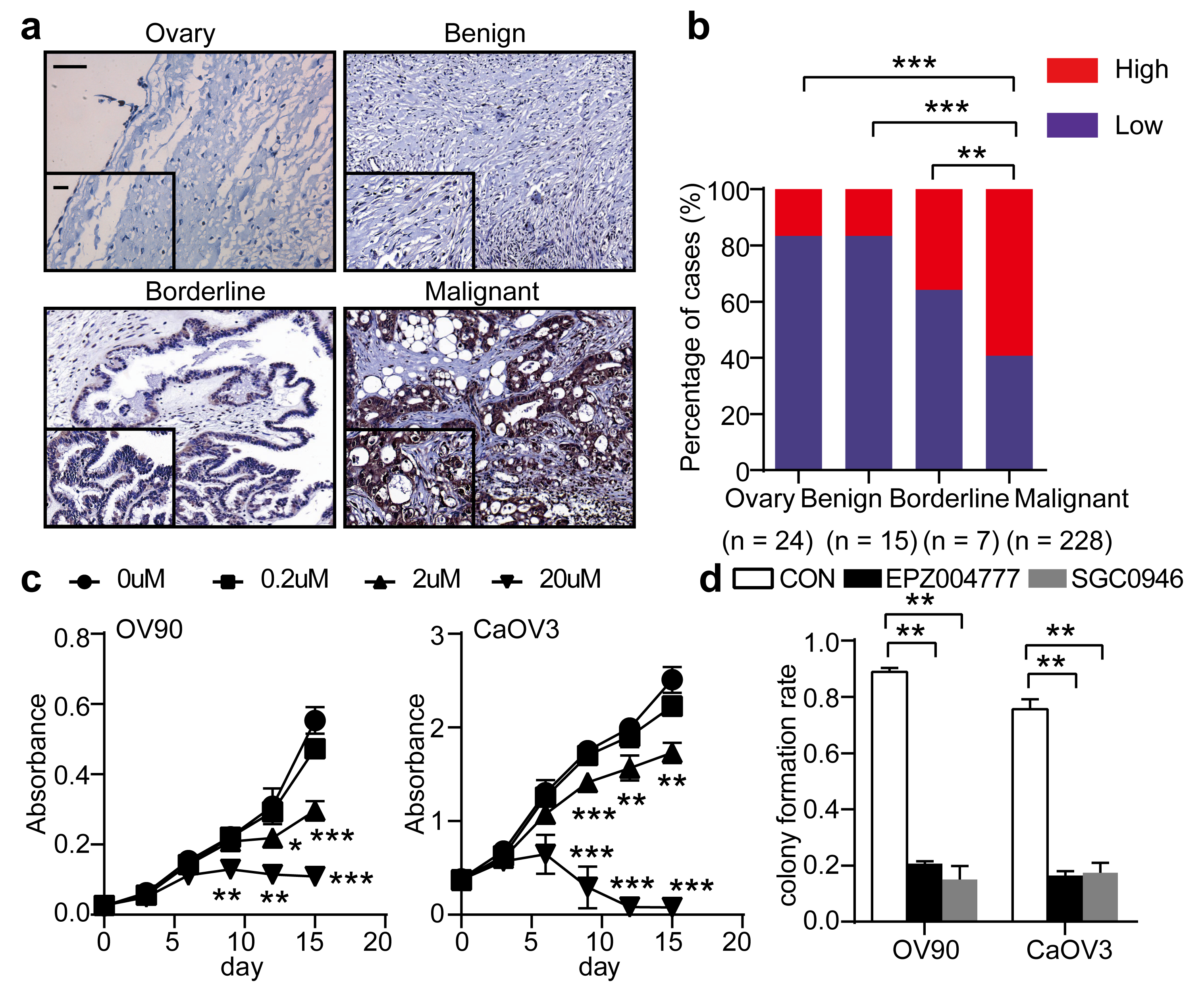

Supplement: Additional file 2: Figure S1. — Immunostaining of H3K79 methylation in human ovarian cancer specimens and effects of DOT1L inhibitors in vitro. (a) Immunohistochemical staining of H3K79 methylation in human ovarian tissues. A brown color in cancer cells is considered as a positive staining. Negative control without first antibody is performed in the normal ovarian tissue. Representative images of H3K79 methylation in normal ovarian tissues and benign, borderline, and malignant ovarian tumors are shown. Original magnification ×100 and ×400. Scale bar 100 μm and 25 μm, respectively. (b) The case rate of H3K79 methylation high and low expression. For comparison between two groups, χ 2 test was applied. (c) OV90 and CaOV3 cells were seeded in 96-well plates and cultured in the presence of SGC0946 at variable concentrations (0, 0.2, 2, or 20 μM) for up to 12 days. Then the OD450 values of corresponding cells were measured at 0, 3, 6, 9, and 12 days after SGC0946 treatment. The results are representative of three independent experiments. (n = 3, P < 0.05). (d) The colony formation assay was performed in OV90 and CaOV3 cells after 10 μM EPZ004777 and 10 μM SGC0946 treatment for up to 12 days. The histogram of three independent experiments was shown. (n = 3, P < 0.05). (TIF 5990 kb) [file 13045_2017_400_MOESM2_ESM.tif]

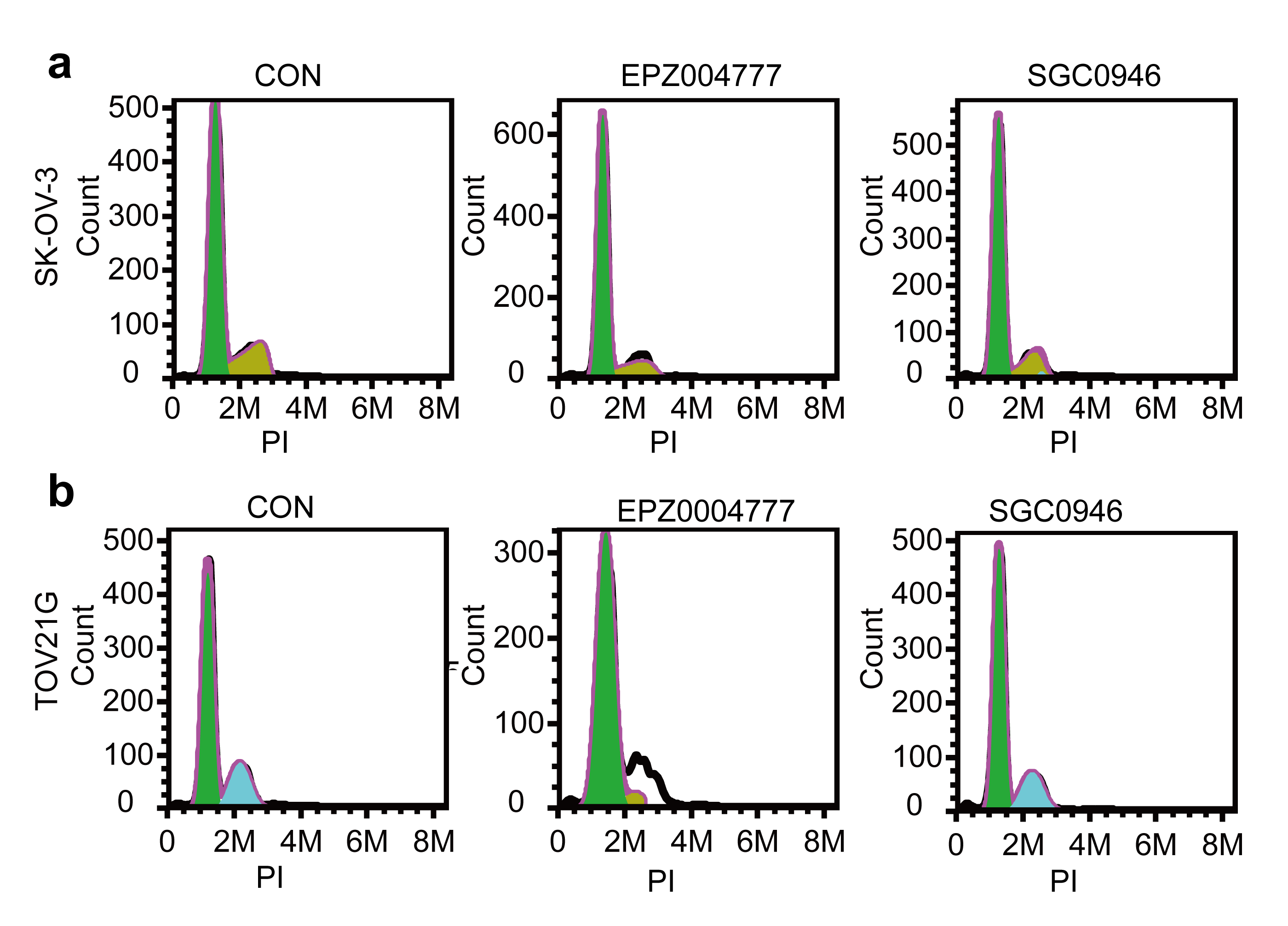

Supplement: Additional file 4: Figure S2. — Effect of blocking DOT1L in cell cycle. After exposure to 10 μM EPZ004777 and SGC0946 seperately for 12 days in both SK-OV-3 and TOV21G cells, cell apoptosis and cell cycle analyses in each group were determined by flow cytometry. The typical images of cell cycle in SK-OV-3 cells (a) and in TOV21G cells (b) were shown. (TIF 713 kb) [file 13045_2017_400_MOESM4_ESM.tif]

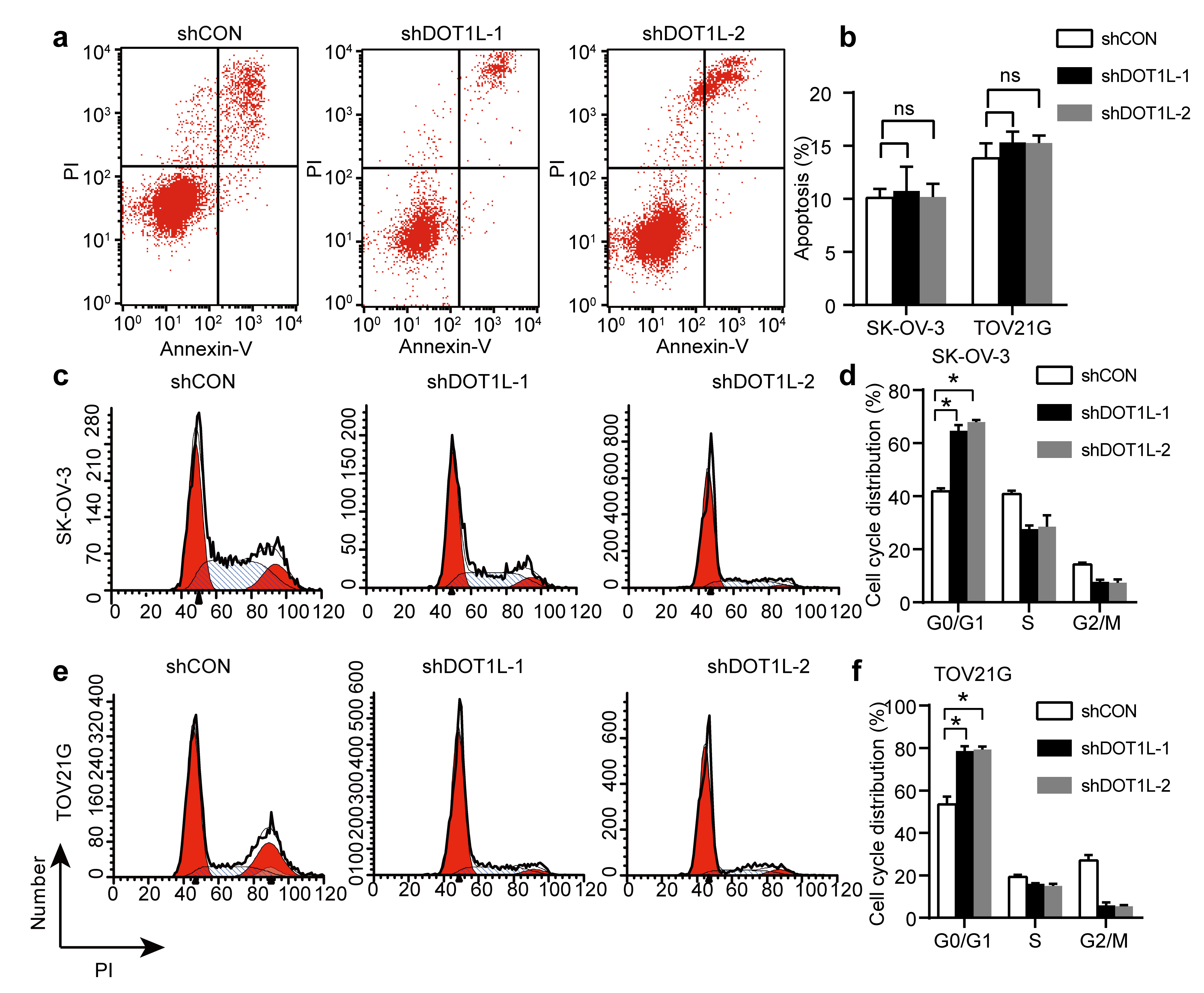

Supplement: Additional file 5: Figure S3. — Effect of blocking DOT1L using shRNAs in the cell apoptosis and cell cycle. ShDOT1L-1 and shDOT1L-2 shRNA lentiviral particles (GeneChem) were used to knock-down DOT1L expression in both SK-OV-3 and TOV21G cells, cell apoptosis and cell cycle analyses in each group were determined by flow cytometry. (a) The typical images and (b) the proportion of apoptosis cell were measured in SK-OV-3 and TOV21G cells. The typical images (c) and the proportion of cell cycle distribution (d) in SK-OV-3 cells were shown. The typical images (e) and the proportion of cell cycle distribution (f) in TOV21G cells were shown. Columns are the mean of triplicate samples. (TIF 1351 kb) [file 13045_2017_400_MOESM5_ESM.tif]

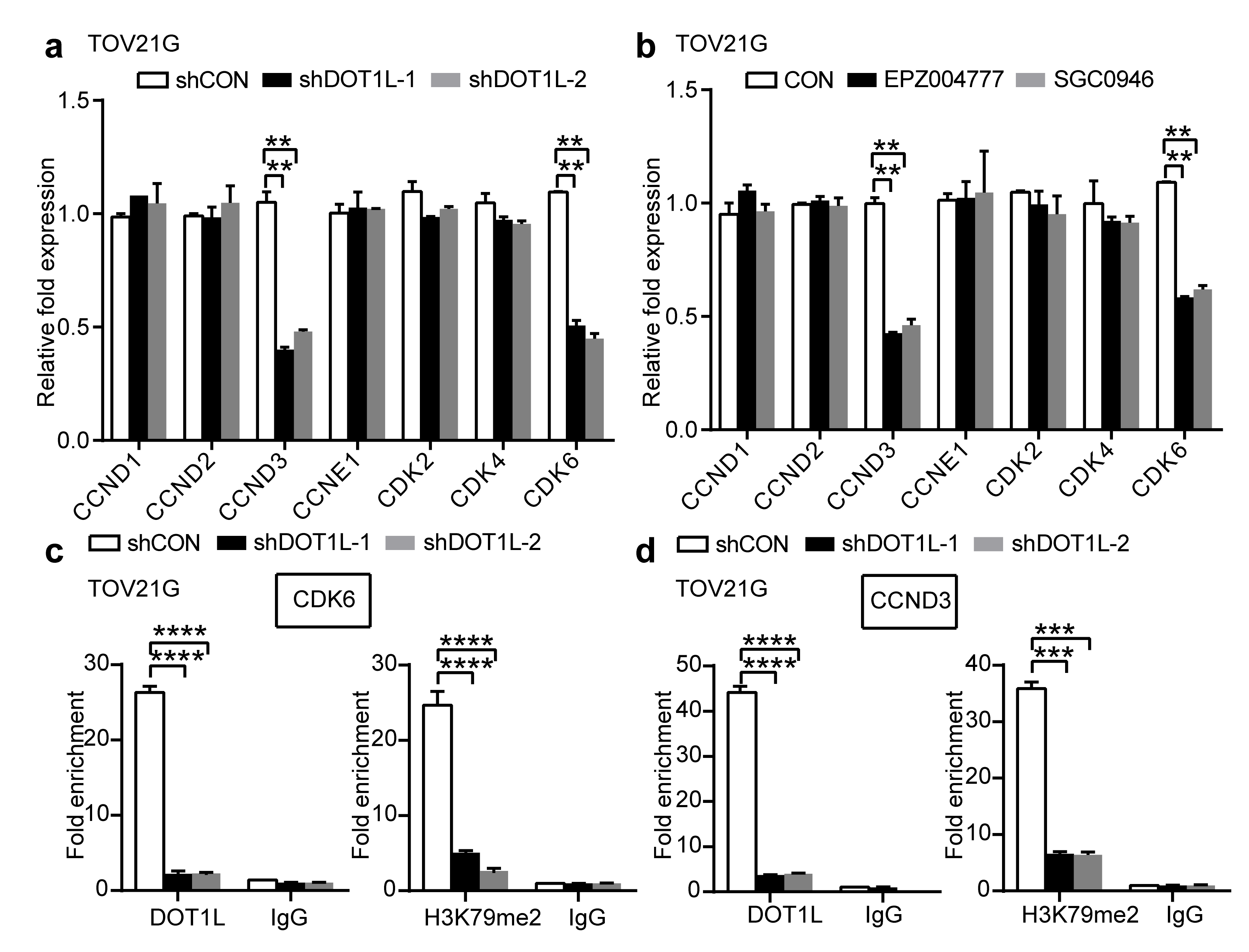

Supplement: Additional file 6: Figure S4. — DOT1L regulates the transcription of G1 arrest genes CDK6 and CCND3 through H3K79 dimethylation. (a) and (b) RT-PCR detecting the change of primary G1 arrest genes, D-type cyclins (D1, D2, and D3), cyclin E, Cdks (2, 4, and 6) in mRNA levels in TOV21G cells knocking down of DOT1L with shDOT1L shRNAs lentiviral particles (a) and DOT1L inhibitors, EPZ004777 and SGC0946 (b) (P < 0.05, Student’s t test, data represented as mean±SD). (c) and (d) DOT1L transcriptionally regulates the expression of CDK6 (c) and CCND3 (d) through induction of H3K79 dimethylation in the binding regions of these genes. Chromatin immunoprecipitation (ChIP) PCR assays were performed in a loss-of-function system. TOV21G cells were transfected with shDOT1L shRNAs and shCON lentiviral particles, and ChIP assays using anti-DOT1L and anti-H3K79me2 antibodies were performed with ChIP primers targeting the binding regions of CDK6 and CCND3 (P < 0.05, Student’s t test, data represented as mean±SD). (TIF 815 kb) [file 13045_2017_400_MOESM6_ESM.tif]
