# Supplementary material for: Prognostic and therapeutic value of disruptor of telomeric silencing-1-like (DOT1L) expression in patients with ovarian cancer
Source: J Hematol Oncol. 2017 Jan 23;10:29. doi: 10.1186/s13045-017-0400-8 (PMC5259947; doi:10.1186/s13045-017-0400-8)
Supplement: Additional file 3: Table S2. — Univariate Cox regression analysis of the clinicopathogical parameters for overall survival and progression free survival. (DOCX 17 kb) [file 13045_2017_400_MOESM3_ESM.docx]

**Additional file 3: Table S2**

**Univariate Cox Regression analysis of the clinicopathogical parameters for Overall Survival and Progression Free Survival**

|  | Overall Survival | |  | Progression Free Survival | | | |
| --- | --- | --- | --- | --- | --- | --- | --- |
|  | HR | 95% CI | *P* |  | HR | 95% CI | *P* |
| FIGO stage (early stage vs advanced stage) | 1.739 | 0.872-3.467 | 0.116 |  | 1.602 | 0.901-2.849 | 0.109 |
| Histologic grade (well vs poor and moderate) | 1.087 | 0.613-1.929 | 0.776 |  | 1.408 | 0.842-2.354 | 0.193 |
| Ascites (no vs yes) | 0.859 | 0.465-1.589 | 0.628 |  | 0.739 | 0.450-1.212 | 0.231 |
| LN metastasis (no vs yes) | 1.253 | 0.773-2.032 | 0.360 |  | 1.199 | 0.802-1.794 | 0.377 |
| Age (≤50 y vs ＞50 y) | 1.762 | 1.160-2.678 | 0.008 |  | 1.291 | 0.911-1.829 | 0.150 |
| Preoperative CA125 (U/ml) (≤600 vs ＞600) | 0.958 | 0.561-1.634 | 0.874 |  | 0.870 | 0.555-1.364 | 0.544 |
| DOT1L expression ( low vs high ) | 3.890 | 2.419-6.258 | 0.000 |  | 3.078 | 2.115-4.479 | 0.000 |

Abbreviations: HR Hazard Ratio, CI Confidence Interval
